# Prevalence of colonisation with third generation cephalosporin-resistant enterobacteriacae (3GCREB) on admission - a cross-sectional study in 6 university hospitals

**DOI:** 10.1186/2047-2994-4-S1-O43

**Published:** 2015-06-16

**Authors:** AM Rohde, J Zweigner, M Wiese-Posselt, A Hamprecht, W Kern, P Gastmeier, H Seifert

**Affiliations:** 1 Charité, Berlin, Germany; 2 University Hospital Cologne, Cologne, Germany; 3 University Hospital Freiburg, Freiburg, Germany

## Introduction

This admission prevalence survey is part of the multicenter study ATHOS (antibiotic therapy optimisation study). ATHOS aims at collecting prevalence and incidence data for nosocomial carriage of multi-drug resistant organisms (MDROs) and to intervene in the inpatient and outpatient setting.

## Objectives

The aim of this admission prevalence survey was to assess the rectal carriage of third generation cephalosporin-resistant enterobacteria (3GCREB) in patients on hospital admission and to perform risk factor analyses for 3GCREB carriage.

## Methods

In 2014, we recruited adult patients within 72 h of admission to non-intensive care units in six German university hospitals. We obtained rectal swabs that were screened for 3GCREB. Each patient was asked to answer a short questionnaire on potential risk factors for colonisation with MDROs. Univariable and multivariable risk factor analyses were performed on preliminary data to identify those factors that were associated with 3GCREB prevalence.

## Results

Of the 4372 patients included, 423 patients were 3GCREB carriers (admission prevalence of 9.7%). Most isolates were *Escherichia coli* (76.8%). Surprisingly, 41.9% of all 3GCREB isolates were additionally resistant to fluoroquinolones. Only two patients (<0.1%) were colonised with carbapenemase-producing enterobacteria. Multivariable analysis associated the following risk factors with 3GCREB colonisation: centre, previous MDRO colonisation (OR = 2.16, p<0.001), antibiotic use (OR=2.08, p<0.001), travel abroad (OR=1.26, p=0.033) and management of gastroesophageal reflux disease (GERD) (OR=1.1, p=0.047).

## Conclusion

To our knowledge, this is one of the largest admission prevalence surveys of 3GCREB in Germany. Interestingly, medical management of GERD and the specific centres to which the patients where admitted proved to be additional risk factors for 3GCREB colonisation on hospital admission. Whether information present on admission will be useful to improve prediction of nosocomial colonisation and infection as well as target infection control measures and therapy needs to be determined.

## Disclosure of interest

None declared.

